# Development and Characterization of Trihexyphenidyl Orodispersible Minitablets: A Challenge to Fill the Therapeutic Gap in Neuropediatrics

**DOI:** 10.3390/pharmaceutics17010005

**Published:** 2024-12-24

**Authors:** Camila Olivera, Oriana Boscolo, Cecilia Dobrecky, Claudia A. Ortega, Laura S. Favier, Valeria A. Cianchino, Sabrina Flor, Silvia Lucangioli

**Affiliations:** 1 Departamento de Tecnología Farmacéutica, Facultad de Farmacia y Bioquímica, Universidad de Buenos Aires, Junin 956, Buenos Aires C1113AAD, Argentina; colivera@docente.ffyb.uba.ar (C.O.); sflor@ffyb.uba.ar (S.F.); 2 Instituto de Tecnología Farmacéutica y Biofarmacia (InTecFyB), Facultad de Farmacia y Bioquímica, Universidad de Buenos Aires, Junin 956, Buenos Aires C1113AAD, Argentina; 3 Consejo Nacional de Investigaciones Científicas y Técnicas (CONICET), Godoy Cruz 2290, Buenos Aires C1414AAD, Argentina; 4 Departamento de Farmacología, Facultad de Farmacia y Bioquímica, Universidad de Buenos Aires, Junin 956, Buenos Aires C1113AAD, Argentina; 5 Área de Farmacotecnia, Ética y Legislación Farmacéutica, Departamento de Farmacia, Facultad de Química, Bioquímica y Farmacia, Universidad Nacional de San Luis, San Luis D5700HHW, Argentina

**Keywords:** trihexyphenidyl, orodispersible minitablets, pediatric pharmacotherapy, orphan formulation

## Abstract

**Background:** Trihexyphenidyl (THP) has been widely used for over three decades as pediatric pharmacotherapy in patients affected by segmental and generalized dystonia. In order to achieve effective and safe pharmacotherapy for this population, new formulations are needed. **Objective:** The aim of this work is the development of trihexyphenidyl orodispersible minitablets (ODMTs) for pediatric use. **Methods:** Six different excipients were tested as diluents. The properties of powder mixtures were evaluated before direct compression and pharmacotechnical tests were performed on the final formulation. The determination of the API content, uniformity of dosage, and physicochemical stability studies were analyzed by an HPLC-UV method. **Results**: The developed ODMTs met pharmacopeia specifications for content, hardness, friability, disintegration, and dissolution tests. The physicochemical stability study performed over 18 months shows that API content remains within 90.0–110.0% at least for this period. **Conclusions:** These ODMTs will allow efficient, safe, and high-quality pharmacotherapy.

## 1. Introduction

**Figure 1:**
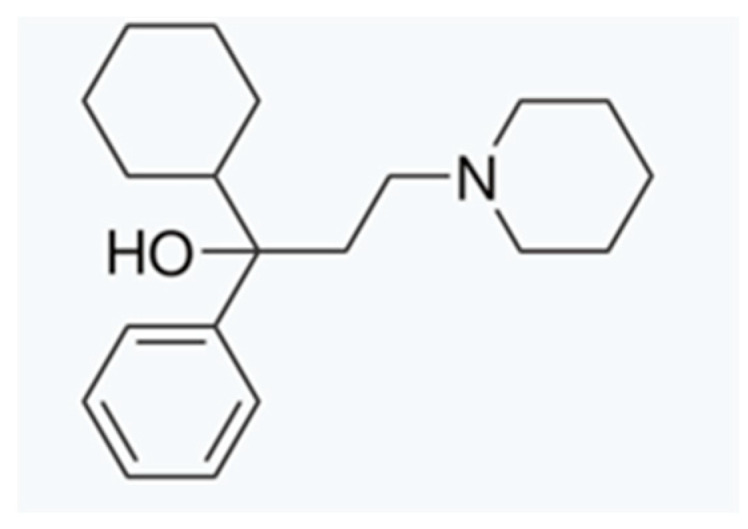
Trihexyphenidyl chemical structure.

Trihexyphenidyl (THP) (1-cyclohexyl-1-phenyl-3-piperidin-1-ylpropan-1-ol) (Figure 1) [1] acts as an anticholinergic agent by intervening at the level of muscarinic receptors. While its most popular therapeutic use is for treating Parkinson’s disease in adult patients, it has been widely used over three decades as pediatric pharmacotherapy in patients affected by segmental and generalized dystonia [2,3]. This neurological condition is characterized by sustained or intermittent muscle contractions, causing anormal movements or postures [4] that can be painful. The contractions they suffer from impact on patient autonomy and movement capacity, also affecting speech and participation in daily activities [5].

THP is used as pharmacotherapy in children with extrapyramidal cerebral palsy (PCEP), where a variety of involuntary movements predominate, and it is characterized by postural instability secondary to defective regulation of muscle tone and coordination [6]. In this type of pathology affecting children, oral administration formulations are commonly used [7,8] and are considered preferable whenever possible. The pharmacokinetics and pharmacodynamics of the active substance differ between children and adults due to differences in their metabolic development. Therefore, when developing a formulation for pediatrics, dosing flexibility as well as easy administration and patient acceptability must be considered [9].

Hence, it is necessary to develop administration systems appropriate to the patient’s age range while maintaining their safety, efficacy, and accessibility. In most cases, available conventional oral formulations are not intended to meet the requirements of this subgroup of the population [10]. Thanks to scientific and technological advances, new pharmaceutical forms have emerged with advantages in terms of administration convenience, dosing, and efficacy. Minitablets are relatively new solid pharmaceutical forms that present the advantages of conventional tablets and, additionally, are associated with ease and flexibility of dosing. Clinical studies demonstrate that the use of THP has improved symptoms associated with dystonia [2], which often manifest early in life.

The World Health Organization (WHO) and international regulatory agencies work to raise awareness and promote activities related to pediatric research in both academic and industrial settings [11].

In pursuit of achieving effective and safe pharmacotherapy in the pediatric population, the development of new formulations that meet their needs is required. The therapeutic requirements of pediatric patients, unlike adults, continuously change as their organism does [12]. Ensuring patient adherence to treatment is critical. Due to the swallowing difficulty that pediatric patients have, liquid formulations are usually prescribed, but the physicochemical and microbiological instability they present must be considered, as well as their high transportation and storage costs.

Orodispersible minitablets (ODMTs) emerge as a response to pediatric pharmacotherapy since, due to their small size and rapid disintegration, they are ingested without any difficulty [13,14,15]. They are defined as tablets with a diameter smaller than 3 mm [16] which, once placed in the oral cavity, disintegrate within a few seconds upon contact with saliva [17].

Processed and co-processed excipients are widely used in the development of orodispersible tablet formulations. After undergoing a co-processing technique, such as spray drying, the new excipients present advantages over their individual previous properties. Among them may be found particle shape and size uniformity, increased porosity, and decreased sensitivity to lubricants, improving flowability and compressibility [18]. These types of excipients can encompass diluents as well as superdisintegrants, glidants, and binders. A distinction should be made between different types of co-processed excipients based on their chemical characteristics. On one hand, there are those based on lactose, mannitol, sorbitol, and starch, and on the other hand, there are those based on cellulose such as microcrystalline cellulose and other derivatives. In this work, six different processed and co-processed excipients were tested for potential use in producing ODMTs by direct compression. There are databases that compile toxicological as well as clinical information about the use of different excipients in the pediatric population. The processed and co-processed excipients tested are widely used in the production of various pharmaceutical forms.

Pediatric patients with dystonia associated with cerebral palsy should start pharmacotherapy with a dose of 0.02–0.06 mg/kg of THP administered two to three times a day (up to a dose of 1–2 mg) with weekly increments of 0.05–0.1 mg/kg [19,20,21].

In order to fill the gap in pediatric therapeutics, THP ODMTs have been designed, developed, and characterized in response to the absence of pediatric formulation of the API in some pharmaceutical markets [22,23]. There is no literature yet describing the development of solid THP formulas specifically targeted at this population subgroup.

Currently, there is no available solid form of THP ODMTs destined for the pediatric population. The aim of this work was the development and characterization of an orphan formulation, as it is based on a known and studied drug but not commercially available in an appropriate preparation or dose [24].

## 2. Materials and Methods

### 2.1. Chemical and Reagents

Trihexyphenidyl hydrochloride (100.79 DB, MW 337.93) and magnesium stearate were purchased from Parafarm, Saporiti Drogstore, Buenos Aires, Argentina. StarLac^®^ was acquired from Meggle Pharma, Wasserburg am Inn, Germany; and Disolcel^®^ from Mingtai Chemical Co., Ltd., Taoyuan, Taiwan. Pharmaburst^®^ was purchased from Unifarma s.a, Buenos Aires, Argentina; Mannogem 2080^®^ from SPI Pharma, Wilmington, DE, USA; Cellactose 80^®^, Avicel C15^®^ from FMC BioPolymer, Philadelphia, PA, USA; and Avicel RC 591^®^ from Productos Destilados SAICyF, Buenos Aires, Argentina.

### 2.2. Equipment

Physicochemical Stability Study was performed working with an HPLC-UV (Thermo Fisher Scientific Vanquish HPLC) coupled to a diode array spectrophotometer detector (Waltham, MA, USA) using an XBridge^TM^ C18 (5 μm, 4.6 × 150 mm) Hypersil GOLD C18 column. Chromatographic conditions were 30 °C column temperature, 20 µL injection volume at 215 nm, with a flow rate of 1.0 mL/min. The mobile phase consisted of methanol: 1% p/v ammonium acetate pH 6.5 (75:25) according to a method previously developed and validated in house.

### 2.3. ODMTs

#### 2.3.1. Design and Preformulation Study

A direct compression technique was used for the development of THP ODMTs. The powder mixture was compressed using a SC-1 mini-press single-punch eccentric machine (Talleres Sanchez, Ciudad Autónoma de Buenos Aires, Argentina) tablet press with a 3 mm diameter round-shaped punch. The direct compression pathway was selected for the manufacture of the ODMTs since it involves the lowest number of steps in the production process that could be easily implemented in hospital facilities. ODMTs were characterized by determining their organoleptic properties, as well as pharmacotechnical parameters such as weight uniformity, disintegration time, friability, and hardness. The identification, quantification, and dose uniformity of the THP ODMTs were carried out through an HPLC-UV.

The requirements of dosage forms for the pediatric population include the use of as few excipients as possible in the final formulation. There are excipients in which a co-processing method is carried out in order to improve the properties of the individual excipients. The resulting co-processed excipients will have improved flowability and compressibility properties, as well as reduced moisture and lubricant sensitivity, improving tableting performance. The selection of the most suitable co-processed excipient is a critical point in the development and manufacture of ODMTs.

For the development of the ODMTs, six different excipients have been tested as diluents of the final formulation. After a search of the literature and market research for the most common excipients used in tablet production, with established safety, six co-processed excipients have been selected to evaluate rheological and pharmacotechnical parameters. The co-processed diluents are mentioned below: lactose/starch, fructose/starch, mannitol/sorbitol, lactose/cellulose, microcrystalline cellulose/guar gum, and microcrystalline cellulose/sodium carboxymethylcellulose [25].

For preformulation studies, characterization of the physical mixture has been carried out. Powder rheological properties such as angle of repose, bulk and tapped density, Carr’s index, and Hausner ratio were determined in each powder blend. To evaluate drug-excipients physicochemical compatibility, FTIR analysis and scanning electron microscopy (SEM) studies were performed.

Once the best co-processed and active pharmaceutical ingredient (API) mixtures in terms of flowability, compressibility, and compatibility were defined, the final qualitative/quantitative formulation was optimized in terms of percentage of superdisintegrant, lubricant, and glidant.

The diluent/API ratio in the powder blend was evaluated. To verify the non-interaction, an estimated proportion of the rest of the excipients present in the final formulation was used (superdisintegrant, lubricant, and glidant). The blending was made by sieving the powders through a 35-mesh sieve and using a tumbling mixer (Erweka V mixer model AR 403), achieving powder homogeneity after 10 min of mixing confirmed by sampling analysis and energy-dispersive X-ray spectroscopy. Rheological pre-compression parameters were tested and compared. A codified numerical scale (USP 43) [26] was used to determine if the angle of repose, Carr’s index, and Hausner ratio were bad, acceptable, good, very good, or excellent for each case.

#### 2.3.2. Flowability, Density, and Compressibility Parameters

##### Angle of Repose (°)

Powder flowability was determined according to USP-43 [26] by calculating the angle of repose, where the blend is allowed to fall freely through a funnel at a specific height and size. The height of the funnel was adjusted in such a way that the bottom of the funnel just touched the tip of the powder. The angle of repose is determined by the horizontal plane and the inclination of the resulting cone, and its numerical value is indicative of powder flowability. The diameter and height of the powder cone was measured. This is a critical parameter in the process of tablet direct compression, especially when manufacturing tablets with a 3 mm diameter punch.

Angle of repose was determined by the following equation:

tan θ = h/r

where θ is angle of repose, h is the height, and r is the radius of the cone.

##### Bulk and Tapped Density

A powder blend was accurately weighed and placed in a 100 mL measuring cylinder using an Erweka-D63150. Bulk density (Bd) is determined by the following formula:

Bd = powder weight/powder volume

The determination of the tapped density (Td) is calculated in the same way as powder bulk density by determining the volume at 1250 and 500 taps, setting a speed of 300 taps/min according to USP method I [26] with a powder density tester. The following formula is used for calculation:

Td = powder weight/tapped powder volume

##### Hausner Ratio (Hr)

The Hausner ratio (Hr) is defined by the following formula and provides information on powder flowability.

Hr = tapped density (V_0_)/bulk density (V_f_)

##### Carr’s Index (%)

Carr’s index (CI) is related to the Hausner ratio as both provide powder compressibility characteristics.

CI (%) = 100 × [(V_0_ − V_f_)/V_0_]

Each rheological parameter evaluated was determined in triplicate, and the average of each one was considered for choosing the best diluent that meets the necessary requirements and properties to perform direct compression.

#### 2.3.3. Pharmaceutical Formula Optimization

For the development of the THP ODMTs, the following excipients were selected as part of the final formulation (Table 1): co-processed lactose and corn starch (Star-Lac^®^), magnesium stearate, sodium croscarmellose (DISOLCEL^®^), and colloidal silicon dioxide (AEROSIL^®^) using the direct compression (DC) method. Different proportions were tested in different combinations once the chosen diluent was defined. For the first stage, the final formula was based on the minitablets’ pharmacotechnical properties in terms of hardness, friability, and disintegration.

Sodium croscarmellose, magnesium stearate, and silicium dioxide were incorporated as pharmaceutical excipients that provided superdisintegrant, lubricant, and glidant/adsorbent characteristics to the formulation, respectively. The proportion of sodium croscarmellose and silicium dioxide was evaluated, while magnesium stearate was established according to bibliographic data.

To optimize the mixing time, a sample collection was made at 4 different times by quarts. These samples were processed and analyzed by an HPLC-UV with a previously developed and validated method, and the RSD between the samples for each time was calculated. An RSD value of less than 2% suggests that the blend was homogeneous. Lubricant mixing time is a crucial parameter when magnesium stearate is used. Due to its hydrophobic properties, if the mixing time is not optimized, it could produce a coating layer over the active ingredient for excessive mixing that affects disintegration and dissolution time. Therefore, the lubricant mixing point was established at 1 min. Both parameters are fundamental when it comes to ODMTs.

#### 2.3.4. Compatibility Study Confirmation

##### Attenuated Total Reflection–Fourier Transform Infrared Spectroscopy Analysis (ATR FTIR)

To evaluate API/pharmaceutical excipients’ compatibility, an ATR FTIR analysis was carried out using a Nicolet iS50 spectrometer (Thermo Scientific, Waltham, MA, USA) with a one-reflection diamond crystal with 64 scans at a resolution of 4 cm^−1^. This method allows a simple, fast, and efficient evaluation of possible physical interactions with no need for any previous sample preparation where a mechanical clamp is used to improve the contact between the powder and the equipment. The potential formulation powder mixture was tested by varying the diluent.

##### Scanning Electron Microscopy and Energy-Dispersive X-Ray Spectroscopy

Additional information related to possible incompatibilities is obtained by performing SEM on the API and API/excipient powder mixture using an FEI INSPECT s50 thermionic microscope operated between 0.1 kV and 30 kV. Images were obtained with an ETD (Everhart–Thornley detector) secondary-electron detector and a vCD backscattered electron detector. EDS analyses were obtained using an Octane Pro detector manufactured by EDAX (Pleasanton, CA, USA). The voltage used was 20 kV for EDS analysis. SE and BSE low-voltage imaging, i.e., near 5 kV, was used to avoid the use of Au or C sputtering. An aliquot of raw sample powder was sprinkled on the carbon tape for sample treatment.

By characterizing the morphological surface of the samples and by combining the mentioned techniques, useful information of polymorphic and crystalline habit changes that may occur can be obtained. Since THP has nitrogen in its chemical structure, nitrogen elemental distribution and mapping was carried out combining SEM with energy-dispersive spectroscopy. The homogeneity of the mixture can also be confirmed with additional studies performed using energy-dispersive X-ray spectroscopy.

### 2.4. Production of ODMTs

THP ODMTs were elaborated using a co-processed diluent (Star-Lac), sodium croscarmellose, silicium dioxide, and magnesium stearate by a direct compression technique. All mentioned excipients were accurately weighed and properly mixed, passing first through a No. 40 and 60 sieve for the lubricant. The direct blend characterization and mixing time were previously determined and optimized. Compression was carried out using a single 3 mm punch tablet press. The compression parameters were adjusted to obtain an ODMT batch of approximately 20 mg/minitablet weight.

### 2.5. ODMT Characterization

#### 2.5.1. Pharmacotechnical Parameters

##### Weight Uniformity

Twenty ODMTs were randomly selected and individually weighed. The results were expressed as weight average and RSD.

##### Hardness

Twenty ODMTs were randomly selected, and hardness was evaluated using a YD-1 tablet hardness tester (Tianjin Guoming Medicinal Equipment Co., Ltd., Tianjin, China). The results were expressed as force average (N) and RSD.

##### Friability

Twenty ODMTs were randomly selected and weighed before and after placing them in the CS-4 Friabilometer USP (CS-4 Tablet Friability Tester, Tianjin Guoming Medicinal Equipment Co., Ltd., Tianjin, China) after 100 rotations at 25 rpm.

The percentage weight loss is calculated with the following formula:

%F = (W_initial_ − W_final_)/W_initial_) × 100

where F is for friability measurement.

##### Disintegration Time

Six ODMTs were randomly selected and placed in 900 mL of distilled water maintained at 37 ± 0.5 °C. This test was carried out using a BJ-1 Disintegration Tester (Tianjin guoming medicinal equipment CO., Ltd., Tianjin, China), and the time average and RSD were calculated.

##### Wetting Time

Six ODMTs samples were each placed into a Petri dish over a colored paper filter. The time it takes for the colored solution to reach the upper part of the tablet is recorded as the wetting time. This provides important information relating to tablet disintegration.

#### 2.5.2. In Vitro Dissolution Study and Dissolution Profile

The dissolution test and dissolution profile of the developed THP ODMTs were carried out using USP-43 [26] ERWEKA apparatus I (DT 126 light Series equipment, Frankfurt, Germany) at 100 rpm. The assay was adapted for the evaluation of ODMTs using four minitablets per basket, and 500 mL of acetate buffer pH 4.5 dissolution medium was placed in each vessel and the temperature maintained at 37 ± 0.5 °C. To perform the dissolution assay, an aliquot was withdrawn after 45 min and subsequently quantified. The amount of THP should be no less than 75% according to the USP 43 API monograph [27]. Additionally, the dissolution profile was determined, and aliquots were withdrawn at different times (1, 20, 30, 50, 60, 75, 90, 105, and 120 s and 4, 6, 8, 15, and 45 min) and subsequently quantified [28,29]. In all cases, quantification was carried out by HPLC-UV.

#### 2.5.3. Physicochemical Stability Study

The physicochemical stability study was evaluated by determining the API content and dose uniformity (at time 0). Samples were kept at 25 °C ± 2 °C and at a relative humidity of 60% ± 5% (RH%). The ODMT content was analyzed using an HPLC-UV at 0, 3, 6, 9, 12, and 18 months. All determinations were made in duplicate.

## 3. Results and Discussion

### 3.1. ODMT Compounding

#### 3.1.1. Design and Preformulation Study

There are currently no commercialized THP ODMTs. Suitable THP formulations for children are only found in some countries as liquid formulations (syrup). Recent studies show that young children prefer conventional minitablets to liquid formulations [30]. Acceptance evaluation performed in healthy adults demonstrates ODTs as the preferred dosage form for a given API [31,32]. A randomized controlled crossover trial in healthy volunteers compared conventional tablets, ODTs, and OD minitablets, and the latter were selected for being the easiest to take [33]. All the above demonstrate the benefits of developing this type of formulation as a novel drug delivery system for targeted populations.

The DC technique was used as the method of choice for the manufacture of the minitablets as only a few simple processes are involved, which makes it possible to transfer this technology to hospitals. Although direct compression has advantages compared to other compression techniques, when a small diameter punch is used, other considerations must be taken into account to achieve a successful product. Excipients with very good flowability that allow uniform API distribution are required. For this purpose, direct compression co-processed excipients were tested. These have improved quality characteristics compared to the excipients from which they are derived. Using spray drying to co-process both excipients, synergism is achieved, and a new diluent with fast disintegrating properties and good flowability is obtained. In this sense, favorable attributes of each excipient are maintained and improved after the process.

Excipients used in the manufacture of ODMTs must meet certain additional requirements that include good solubility, rapid disintegration without any residue, pleasant taste, and good mouthfeel.

As well as in common orodispersible tablets (ODTs), a diluent, disintegrant, and lubricant must be included in the formulation. Sodium croscarmellose was chosen as a superdisintegrant, silicium dioxide as glidant, and magnesium stearate as lubricant. To choose the best diluent for the development of ODMTs, six co-processed excipients have been tested.

#### 3.1.2. Flowability, Density, and Compressibility Parameters

As previously explained, when using a 3 mm punch to produce minitablets, it should be ensured that the powder mixture presents excellent pre-compression parameters. Since the diluent has the highest proportion in the formulation, its flowability and compressibility properties must be considered as the determining factor in the production process.

**Figure 2:**
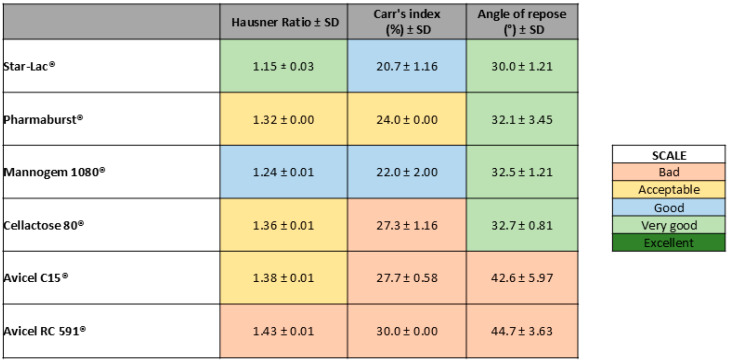
Color scale according to powder rheological parameter results obtained for each physical mixture (diluent + API).

For each physical mixture (diluent + API), three individual measurements were made to determine the Hausner ratio, Carr’s index, and angle of repose. A color was assigned to the average of each parameter and classified as non-acceptable, acceptable, good, very good, or excellent. The results of each parameter according to the USP 43 numerical range [26] scale are color-coded and presented in Figure 2.

As shown in Figure 2, the blend powder containing Star-Lac as the diluent was the one with the best pre-compression parameters obtained.

#### 3.1.3. Pharmaceutical Formula Optimization

The final formulation of the THP ODMTs contains as few market-tested excipients as possible in order to ensure its safety. The selected superdisintegrant is commonly used in tableting and allows the minitablet to disperse immediately upon contact with saliva. Sodium croscarmellose is a cross-linked compound of sodium carboxymethylcellulose, which, through its mechanism, allows the tablet to swell and disintegrate rapidly, promoting the contact of liquid/saliva with the formulation. For orodispersible formulations, it is typically used in a range of 2–5%. Different percentages of sodium croscarmellose have been evaluated. Tablet disintegration and wetting time, as well as tablet hardness and friability, have been taken into account to decide the most effective amount of superdisintegrant to be added in the final powder mixture. Three tablet batches have been elaborated with 2.0, 3.5, and 5.0% of superdisintegrant, respectively (Table 2). In terms of wetting and disintegration time, the batch containing 5.0% of sodium croscarmellose presents a lower time value with respect to the batch containing 2.0% and 3.5%. Each test was carried out in sextuplicate on all the batches prepared (*n* = 6).

The average of these parameters among batches did not show a significant difference and were within the established ranges. Thus, a concentration of 5% of sodium croscarmellose was used in the manufacture of ODMTs. Rapid disintegration and tablet size are the most important parameters in order to deliver a solid form to pediatric patients, ensuring treatment adherence and easy administration.

The proportion of magnesium stearate was established according to a literature search, where 1.0% was the common quantity used, which allows the clean release of the tablet without cracking and reduces the friction between the metal surface and the solid dosage form. As a good mixing practice, the lubricant was incorporated as a final step of the mixing of the powder. Due to its hydrophobic characteristics, a long lubricant mixing time would generate a hydrophobic layer over the API that could negatively affect its dissolution time, delivery, and absorption and consequently influence the treatment effectiveness.

In the case of silicon dioxide, a range between 0.5, 1.0, and 1.5% was tested by evaluating its blend powder pre-compression parameters in the other three batches (Table 2). The amount of 1.0% was set as the minimum that gave the best blend flowability to the mixture.

**Figure 3:**
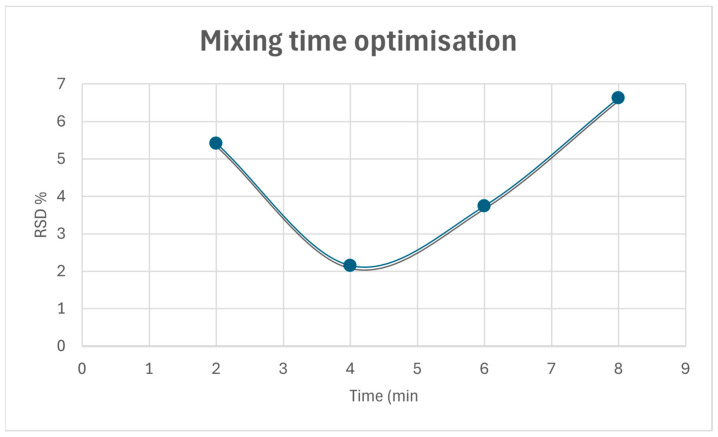
Mixing time optimization.

It is known that the effect of powder mixing time can directly impact on the homogeneity of the blend, as well as the disintegration and dissolution time. A poor homogeneity of the mixture refers to a poor distribution of the API that can ultimately affect the patient’s treatment. Therefore, the uniformity of the mixture must be ensured, and for this reason, a mixing time test was carried out applying the quartering technique. In order to optimize the mixing time, the powder mixture was divided into four equal fractions at different times, and the RSD of the API concentration among these was evaluated. Figure 3 depicts that at low mixing times, the calculated RSD is high. As mixing continues, the RSD value reaches a minimum. From this point on, the increase in the calculated RSD values is indicative of powder heterogeneity. The selected mixing time corresponds to the lowest point of the graph obtained from RSD vs. mixing time.

**Figure 4:**
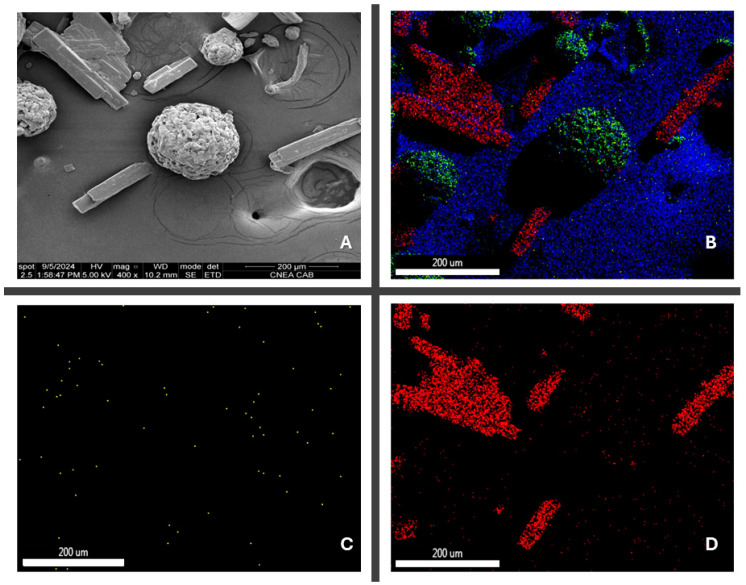
(**A**) Contrast image API/excipient blend 400×; (**B**) API/excipient blend elemental mapping; (**C**) nitrogen mapping; (**D**) chloride mapping.

The images obtained from the elemental composition (Figure 4) show a uniform distribution of the API, which can be confirmed by the distribution of nitrogen and chloride (the only source of which is THP) throughout the area analyzed.

#### 3.1.4. Compatibility Studies

As a preliminary step, different screening methods have been employed on the API, excipients, and powder mixtures. Once ATR-FTIR on all samples was carried out, rheological data were evaluated, and the best API/excipient blend was selected.

To complement the preliminary data and confirm the non-interaction between the excipients of the selected mixture, additional studies employing SEM analysis have been carried out.

##### Attenuated Total Reflection–Fourier Transform Infrared Spectroscopy Analysis

The excipients were tested alone, and in each mixture, the IR spectra obtained were analyzed for possible interactions.

**Figure 5:**
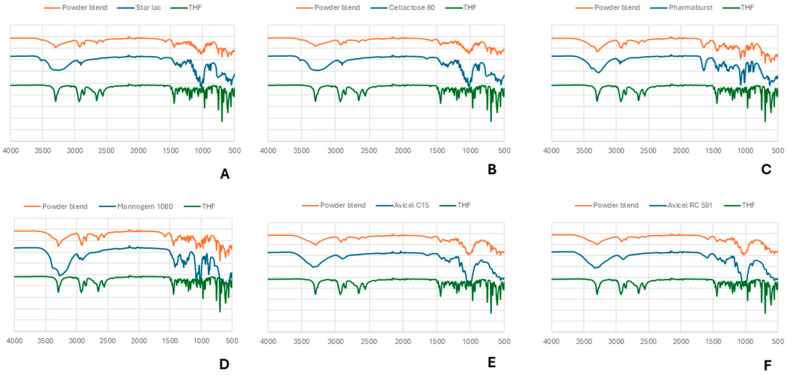
ATR-FTIR analysis of THP and its respective co-process excipient and powder blend. (**A**) Star Lac; (**B**) Cellactose 80; (**C**) Pharmaburst; (**D**) Mannogem 1080; (**E**) Avicel C15; (**F**) Avicel RC 591.

THP characteristic functional peaks at 3294 cm^−1^ (for OH stretching), 2926 cm^−1^ (for aromatic C-H stretch), 969 cm^−1^ (for C-N), and 698 cm^−1^ (for C-H bending) are observed. The characteristic peaks present in the individually obtained spectra are found at the same wavelengths in the excipient’s mixture as a sum of spectra (Figure 5), indicating that there is no interaction between excipients and the active ingredient.

##### Scanning Electron Microscopy and Energy-Dispersive X-Ray Spectroscopy

**Figure 6:**
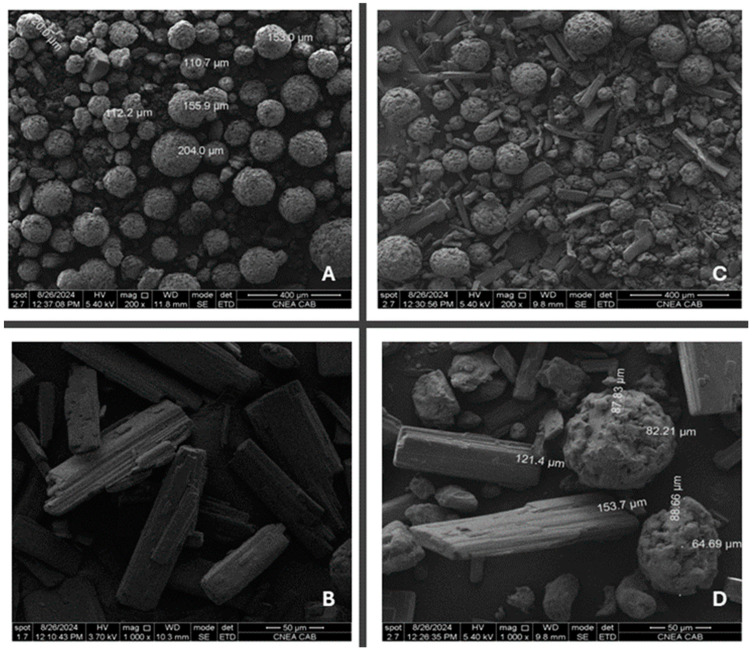
Scanning electron micrographs of (**A**) Star-Lac, (**B**) THP, (**C**) API/excipient blend 200×, and (**D**) API/excipient blend 1000×.

Figure 6 depicts the surface morphology of THP ODMTs. Figure 6A shows that the selected diluent has a spherical shape, which makes it an excellent DC excipient. A detailed distribution of the API and excipient can be observed in Figure 6C,D, where there is no significant difference between their particle sizes, which corresponds to the short mixing time needed to achieve mixture homogeneity. Conversely, a difference in their morphology can be observed, which could explain the segregation of the excipients at longer mixing times.

### 3.2. ODMTs Characterization

#### 3.2.1. Pharmacotechnical Parameters

Due to the absence of international guidelines for ODMTs, requirements for conventional tablets were selected instead. During the production of THP ODMTs, pharmacotechnical tests such as weight uniformity, hardness, friability, and disintegration were performed according to USP-43 [26]. The disintegration time was less than 30 s. This is partly due to the presence of sodium croscarmellose, as previously mentioned, which favors the entry of liquid, causing it to swell thus facilitating disintegration. This result is consistent with previously published work on ODTs containing sodium croscarmellose [34].

Conventional THF tablet formulations show higher disintegration times, ranging from 54 s to 1.23 min [35].

There is no consensus regarding disintegration time limits for ODTs. While the FDA guideline for ODTs recommends a disintegration time of less than 30 s, the European Pharmacopeia states less than 180 s [36]. USP ODT monographs show disintegration times between 30 and 60 s, but there are no established limits for ODTs [26]. The developed formulation shows disintegration times that comply with the more stringent criteria.

The short wetting time obtained is closely related to disintegration time. A summary of pharmacotechnical parameters is shown in Table 3.

#### 3.2.2. In Vitro Dissolution Study and Dissolution Profile

**Figure 7:**
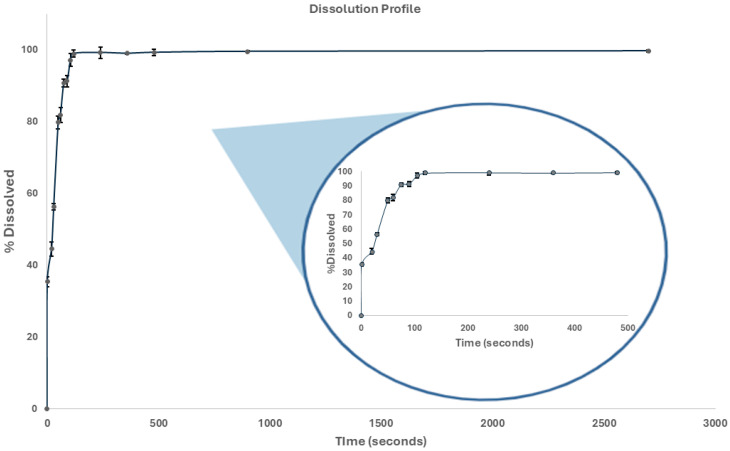
THP ODMT dissolution profile.

The dissolution profile of the THP ODMTs provides information on how the formulation will perform in vivo. As shown in Figure 7 (percentage dissolution vs. time plot), it was observed that 2 min after the start of the test, 100% of the THP was dissolved (Q = 70% according to USP 43 [37]).

#### 3.2.3. Physicochemical Stability Study

A physicochemical stability study was performed over the course of 18 months. Content (as shown in Table 4) and THP dose uniformity remain within USP specification (90.0–110.0% of the labeled value) [37], and content uniformity values are within the established range (85.0–115.0% of the labeled value) and have an RSD of less than 2% [38]. Our findings suggest that the optimized formulation is physically and chemically stable over the studied period.

## 4. Conclusions

The powder mixture obtained using Star-Lac as the co-process diluent was the one that showed the best pre-compression results. The final formulation complies with API content and post-compression parameters (average weight, hardness, friability, and disintegration time) according to pharmacopeial guidelines.

In conclusion, a novel formulation of direct compressed THP ODMTs intended for pediatric administration was successfully designed, developed, optimized, and characterized, and within 18 months, it was physiochemically stable. This will provide the sole treatment alternative for segmental and generalized dystonia in pediatric patients due to the lack of a pharmaceutical formulation available in the market.

## Figures and Tables

**Table 1:** Qualitative–quantitative final formulation.

Function	%	Raw Material
API	12.50	Trihexyphenidyl
Diluent	80.50	Lactose + corn starch co-process
Superdisintegrant	5.00	Sodium croscaramellose
Glidant	1.00	Silicium dioxide
Lubricant	1.00	Magnesium stearate

**Table 2:** Excipient composition for formulation optimization.

Excipient	Batch 1	Batch 2	Batch 3	Batch 4	Batch 5	Batch 6
Superdisintegrant (%)	2.0	3.5	5.0	-	-	-
Glidant/adsorbent (%)	-	-		0.5	1.0	1.5

**Table 3:** Post-compression pharmacotechnical parameters.

Weight Uniformity (mg ± SD)	Hardness (Newton ± SD)	Friability (%)	Disintegration (s ± SD)	Wetting Time (s ± SD)
20.6 ± 0.6	30.9 ± 4.3	<1	29.5 ± 1.1	<1 min

**Table 4:** Determination of THP ODMTs.

Months	API Content (% ± RSD)	API Content (mg ± SD)
0	96.98 ± 0.1	2.42 ± 0.01
3	98.50 ± 0.2	2.46 ± 0.13
6	104.40 ± 0.1	2.61 ± 0.14
9	105.90 ± 0.1	2.65 ± 0.11
12	100.70 ± 0.1	2.52 ± 0.03
18	102.20 ± 0.1	2.56 ± 0.05

## Data Availability

The data presented in this study are available on request from the corresponding author.
